# My Voice Library: Protocol for Developing Audio and Visual Datasets to Enable Personalized Real-Time Communication for People With Dysarthria

**DOI:** 10.2196/97614

**Published:** 2026-07-08

**Authors:** Petra Karlsson, Andrea Bandini, Michelle McInerney, Hayley Smithers-Sheedy, Annemarie Murphy, Maria Dalmon, Alistair McEwan, Silvia Orlandi

**Affiliations:** 1 Faculty of Medicine and Health, Cerebral Palsy Alliance, The University of Sydney, 88 Mallett Street, Camperdown, NB, 2050, Australia, 61 0447508661; 2 The Biorobotics Institute & the Department of Excellence for Robotics and AI, Scuola Superiore Sant’Anna, Pisa, Italy; 3 KITE Research Institute, University Health Network, Toronto, ON, Canada; 4 Department of Engineering ‘Enzo Ferrari’, University of Modena and Reggio Emilia, Modena, Emilia-Romagna, Italy; 5 Department of Language & Cognition, Faculty of Brain Sciences, University College London (UCL), London, United Kingdom; 6 Heart Centre for Children, Westmead Children’s Hospital, Sydney Children’s Hospital Network (SCHN), Sydney, Australia; 7 Paediatrics & Child Health, School of Clinical Medicine, University of New South Wales (UNSW), Sydney, Australia; 8 Cerebral Palsy Alliance, Allambie Heights, New South Wales, Australia; 9 School of Biomedical Engineering, Faculty of Engineering, University of Sydney, Sydney, Australia; 10 Department of Electrical, Electronic, and Information Engineering “Guglielmo Marconi” – DEI, University of Bologna, Bologna, Italy; 11 Rehabilitation Bioengineering Lab, IRCCS Istituto delle Scienze Neurologiche di Bologna (ISNB), Bologna, Italy

**Keywords:** cerebral palsy, speech impairment, communication, technology, patient and public involvement

## Abstract

**Background:**

Communication is a human right; despite this, children with cerebral palsy and dysarthria experience constraints due to their physical disability, affecting their opportunity to reach their full potential and ability to fully participate in play, school, and socializing. For many, their challenge to produce clear speech that can be understood is interpreted by others as an intellectual impairment. Children with moderate-severe speech impairments such as dysarthria often rely on technology solutions, which can translate text or symbols to speech manually or using a single binary switch, scanning hierarchical switch‑access menus. This is time-consuming, arduous, and 15 to 20 times slower than normal speech. Technology innovations, developed via high-quality datasets of audio-video samples of dysarthric speech, hold the key to build personalized speech-recognition algorithms that allow us to bridge this gap.

**Objective:**

My Voice Library is a repository of high-quality audio and visual datasets of children living in Australia with cerebral palsy who have dysarthria. The children are presented with gamified modules based on words and sentences from the Frenchay Dysarthria Assessment 2 to allow speech and language therapists, engineers. and programmers to develop personalized speech-recognition approaches to enable personalized real-time communication.

**Methods:**

Our ethics and consenting process enables participants (8-18 y) to choose if they want their information to be used for personalized research in addition to general research. The study has been approved by the University of Sydney’s Human Research Ethics Committee (2023/263). Caregivers of the participants provide written informed consent. The results will be disseminated through contributions to international conferences and scientific journals, and they will also be included in students’ theses.

**Results:**

The National Health and Medical Research Council funded the study in April 2021. Data collection commenced in March 2024 and will continue until 99 participants have been recruited, after which the database will remain active. As of April 2026, thirteen of 99 participants have been recruited. Preliminary data analysis commenced in 2026. The results from the preliminary analysis are expected to be published in 2027, with full results to be published once 99 participants have been successfully recruited.

**Conclusions:**

My Voice Library embeds high-quality voice and video data assessments through gamified modules based on the Frenchay Dysarthria Assessment 2 and was designed in partnership with an active patient and a public involvement advisory group. My Voice Library and its associated information governance will facilitate the research use of real-world data where ethical approval and consent are given. My Voice Library enables the collection of voice data in controlled yet naturalistic environments for children, enhancing the clinical relevance and quality of the dataset. The modular and gamified design promotes sustained participant engagement, reduces fatigue, and increases the quantity and diversity of data collected.

## Introduction

Each year, in Australia, 1 in 700 children is born with cerebral palsy [1], the most common physical disability of childhood, characterized by disorders of motor function and posture [2,3]. The motor disorders of cerebral palsy are often accompanied by challenges with perception, sensation, cognition, behavior, and epilepsy, affecting a child’s development and ability to explore, move their hands and feet, learn, and gain independence [2,4]. Motor speech disorders are common, with 50% of children having speech impairments (eg, dysarthria and/or limited speech) [1,5,6]. Dysarthria is characterized by slow, weak, imprecise, and uncoordinated speech movements [7], which range from mild to severe. For individuals with moderate-to-severe dysarthria, their speech is difficult to understand [5] and often becomes more unintelligible over time. Being unable to clearly express oneself is inherently frustrating for all children but is particularly so for many children who have good cognition and receptive language skills [8].

Individual and personalized assistive technology access solutions are imperative for children with significant physical disabilities to be able to communicate, demonstrate their abilities, and learn. For those who cannot use a standard mouse, keyboard, or touch screen, access to computers and speech-generating devices may be achieved through the use of technologies operated through eye gaze and head or limb movements [9]. However, these strategies are 15 to 20 times slower than verbal speech [10].

Speech recognition to control assistive technology is not new. However, off-the-shelf speech-recognition algorithms fail even when dysarthria is mild [11]. To address this challenge, a few research databases with fewer than 20 adult voices with cerebral palsy have been established, but none, that we are aware of, for children below the age of 16 years [12-17]. Furthermore, initiatives such as Project Euphonia (Google Research) [18] and the Speech Accessibility Project are databases under development with the aim of addressing and building databases of speech from adult participants with amyotrophic lateral sclerosis, cerebral palsy, Down syndrome, Parkinson disease, and stroke. Commercial enterprises such as Voiceitt [19] have been developed, but no voice repository is accessible for research and critical technology innovation. High-quality access technology research in children with dysarthria is almost nonexistent. However, in adults with dysarthria, a model to systematically recognize lipreading using video-based approaches has been developed, achieving 76% accuracy on a 1000-word recognition task [20]. Furthermore, new video analysis techniques have been proposed, enabling mouse pointer control via head and facial movements [21] and the automatic determination of functional choices through facial expressions in children with multiple disabilities [22]. Other approaches have combined audio and video inputs for multimodal speech recognition in people with disordered speech, improving recognition accuracy by ~8% compared to audio inputs alone [23]. Research in adults participating in automatic speech-generating research, which can be translated for children, demonstrates that they performed best if they were motivated, had access to technology, and were well supported by carers [14]. This demonstrates that both visual and audio feedback are important to engage the participant while recording.

Technological advances are moving rapidly, with artificial intelligence, virtual reality, augmented reality, and brain-computer interface technologies emerging. With verbal comprehension often exceeding expressive capacities in children with speech impairments [8], and without a means to tap into these abilities, there is an urgent need for technological solutions to bridge this gap [24]. There is potential for breakthroughs and transformed care using innovative technology. However, high-quality datasets are of paramount importance for technology development and evaluation. Furthermore, to contribute data, people with cerebral palsy and clinicians working in the field need an accessible portal that takes advantage of the technological capabilities of online speech assessments, supporting more effective, efficient, and accessible disability services.

This study aims to demonstrate the feasibility, scalability, and practicality of the database as measured by the NASA Task Load Index (NASA TLX) [25], the System Usability Scale (SUS) [26], and using false positive and false negative counts to obtain sensitivity and specificity.

Here, we describe the establishment of My Voice Library, a repository of high-quality audio and visual datasets through which researchers, clinicians, and engineers can share their data and expertise to accelerate scientific discovery and collaboration in cerebral palsy technology innovations, enabling personalized real-time communication. These innovations will allow children with cerebral palsy who have speech impairments to be understood as they speak.

## Methods

### Design

This is a multiple single-case study that adopts an exploratory and iterative process where the primary objective is to establish a gamified repository of high-quality audio and visual datasets (My Voice Library) of individuals 8 to 18 years with cerebral palsy and dysarthria and makes data available for research, which will be used by engineers and programmers to enable personalized real-time communication. Our secondary objectives are to (1) establish an advisory panel of people with lived experiences of cerebral palsy and dysarthria who will lend their expertise to studies using My Voice Library data as research partners; (2) develop a platform with an option to later set up a cost-effective and accessible digital health approach for dysarthria assessment and data collection; and (3) make the dataset from My Voice Library available in a program of biomedical engineering research to trial new technology innovations for real-time communication. The My Voice Library will first be piloted in 9 children with cerebral palsy, and thereafter a further 90 participants will be sought for user testing.

### Patient and Public Involvement

This study includes 1 person with lived experience of cerebral palsy and dysarthria as part of the investigator team who, together with additional parents and young people with cerebral palsy, has provided feedback on the development of the My Voice Library platform, the time and burden of completing the modules, participation information sheet, and consent forms. Additionally, our advisory panel includes people living with cerebral palsy and dysarthria, carers, and researchers in the field. Together with the My Voice Library research team, they have provided valuable ideas, feedback, and important networks. The idea for My Voice Library emerged after multiple conversations with people with lived experience of dysarthria and their challenges in finding a fast and reliable communication device that met their needs. The advisors have ensured that the format and the language of both the orientation materials and the modules themselves, as well as the study information and questionnaires included in the research development address matters important to all stakeholders. Our research team member, who has lived experience of cerebral palsy, participated in a short informational video used for recruitment and informational purposes. This video will be included in all plans to disseminate the study findings (Checklist 1).

### Participant Selection

There is no formal referral process. Caregivers of children aged 8 to 18 years who respond to a recruitment initiative will be screened for eligibility criteria (detailed in Textbox 1). Participants will be recruited through Cerebral Palsy Alliance, Australia, and any disability or consumer organizations in the national or international networks of the investigators and consumer advisors who agree to assist in circulating study information. For example, ethics-approved study information will be circulated through Cerebral Palsy Alliance’s website, X (formerly known as Twitter), Facebook, Australian CP Register, and CP Quest moderated by Cerebral Palsy Alliance.

Textbox 1. **Inclusion and exclusion criteria.**

Inclusion criteria

- Aged 8 to 18 years
- Diagnosis of cerebral palsy
- Identified as having dysarthria
- Based in Australia
- English speaking
- Identified as having functional hearing and vision
- Identified as being able to follow 2-step instructions
- Access to the internet and a laptop or personal computer with a video camera
- Upon setting up their account on My Voice Library, individuals who will pilot the program to evaluate performance and usability of the data collection platform

Exclusion criteria

- Participants who upon signing up to have their voice recorded on My Voice Library do not pass the Screening Questionnaire as per items in the inclusion column
- Participants who upon setting up their account on My Voice Library and where the system recognizes that the audio and/or video quality is too limited for a successful recording

### Consent

Informed assent and consent from children and their parents/caregivers/guardians will be sought. The study participation information and consent will be embedded in a link using REDCap (Research Electronic Data Capture) [27], electronic data capture tools hosted at The University of Sydney, a secure web-based software platform designed to support data capture for research studies, providing (1) an intuitive interface for validated data capture, (2) audit trails for tracking data manipulation and export procedures, (3) automated export procedures for seamless data downloads to common statistical packages, and (4) procedures for data integration and interoperability with external sources. Additionally, within the My Voice Library account setup, consent needs will be reconfirmed.

### Procedures and Outcome Measures

My Voice Library participants are presented with audio-recorded adult models of each word or sentence in gamified modules based on words and sentences from the Frenchay Dysarthria Assessment—Second Edition [28] to allow speech and language therapists, engineers, and programmers to develop new innovations to enable personalized real-time communication.

It is anticipated that it will take 10 to 15 minutes to set up an account with the My Voice Library database. Part of this setup includes information about the child: geographical location, age, diagnosis, level of dysarthria (mild, moderate, or severe if a prior assessment has been conducted), communication impairment (Communication Function Classification System) [29], speech performance (Viking Speech Scale) [30], hearing and vision, language used at home, 3 questions that require the child to follow a 2-step instruction to complete, and confirmation that they have access to a laptop or personal computer with a video camera (Multimedia Appendix 1).

Before the participants start, they will be taken through an onboarding to familiarize themselves with how and what they are expected to do in the game-based modules. The parents of the children will be the main individuals to provide support; however, study personnel will be available when required.

The game-based modules are designed to create an engaging environment for children to record audio and video under the supervision of their caregiver and can be completed within 3 to 4 hours. However, this estimate is highly individual, and participants will be given the option to stop a recording and revisit it at a later stage, where they can pick up from where they left off. It is recommended that participants complete My Voice Library within 2 weeks of starting.

After completing the My Voice Library modules, study personnel will complete 2 questionnaires: the NASA TLX [25] and the SUS [26]. Each questionnaire may take about 10 minutes to complete, totaling 20 minutes. These measures will be completed with the child participant and their caregiver via telepractice/videoconferencing.

Finally, at the end of the user experience surveys, the participants and their caregivers will be invited to enter their contact details into a separate database with the view that future researchers may contact them to invite their participation in studies where they will be able to try out technology developed using audio and/or video data from My Voice Library. This section will take no more than 5 minutes to complete.

### Audio and Video

**Figure 1.**
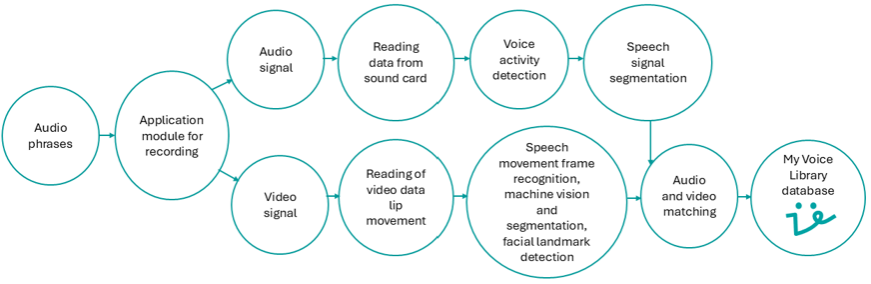
My Voice Library architecture for audio-video database recording.

My Voice Library combines audio and videos associated with the speech sounds of children with cerebral palsy and all severity levels of dysarthria in a creative, progressive web app (Figure 1). All authors were actively involved in the design of the system, providing input and feedback throughout its development. The audio and video recordings of the children’s voices will be set up through a game-based platform (My Voice Library Database Dictionary; Multimedia Appendix 1).

Where possible, video data will be deidentified using a privacy-enabling algorithm, which prevents the face recognition software from reliably recognizing faces while maintaining facial details in the recording [31]. The following details are reported in the participant information statement provided to all participants and their families:

Contact details will be securely stored on REDCap and hosted outside of the My Voice Library under the University of Sydney’s data management. Data stored in the My Voice Library will be hosted by Amazon Web Services. Providing the highest security of data storage, all data will be managed in accordance with The University of Sydney Secure Research Data Store. In order to maintain your/your child’s confidentiality, they will be allocated a unique participant number, and all audio and video data will be stored under a specific participant number. Your/your child’s name will not be stored. Where possible, video data will be deidentified using a privacy-enabling algorithm, before being made available to researchers external to the My Voice Library research team, who hold ethics approval from The University of Sydney’s Human Research Ethics Committee. A Research Data Management Committee will oversee the project.

The children’s speech sounds will be recorded using a digital audio recorder at a 44.1-kHz sampling rate (16-bit quantization) with a microphone integrated into a computer or tablet. Video data of lipreading will be acquired using the built-in webcam of a computer or tablet. Children will hear audio-recorded adult models of each word or sentence within the game and will be asked to repeat the sounds.

### Questionnaires

The questionnaire collected information on participants’ demographic characteristics (birth year, sex, and country); primary motor type of cerebral palsy; classifications of vision, speech, hearing, gross motor, fine motor, communication, eating, and drinking; and payment information.

The SUS [26], which measures the perceived ease of use of software or hardware technology in 10 items, will be utilized. Response efficiency will be assessed using the NASA TLX [25], which measures mental, physical, and temporal demand, as well as performance, effort, and frustration across 6 items and subscales. The graphical 1 to 7 scale, with no weighted scoring, will be used. These tools will be modified for children and administered by study personnel once the My Voice Library modules have been completed. Study personnel will complete this process with children via video conferencing.

### Sample Size

The study uses a multiple single-case design, in which each participant is analyzed individually. The sample size (n=99) was determined pragmatically, consistent with recommendations for feasibility and pilot studies, which is not intended to be powered for hypothesis testing [32,33].

The number of cases was selected to ensure sufficient representation of variability in dysarthric speech characteristics and to support the development of a comprehensive speech database. This approach aligns with current methodological guidance, emphasizing that feasibility studies should include enough cases to inform future research and methodological development. Any subgroup analyses by severity, motor type, age, or gender will be exploratory.

### Data Analysis Plan

Descriptive statistics will be used to describe the dataset of children with cerebral palsy. Feasibility, usability, data quality, and scalability will be evaluated using a combination of quantitative and qualitative indicators. Usability will be assessed using a modified version of the SUS [26], with mean SUS scores interpreted against established benchmarks (scores ≥68 indicating satisfactory usability and ≥70‐80 indicating good usability). Cognitive workload will be evaluated using the NASA-TLX [25], with subscale and overall workload scores analyzed to identify perceived effort, mental demand, and frustration associated with using the system.

Correlations, associations, and odds ratios will be examined using appropriate statistical methods to explore relationships between participant characteristics and outcomes on the SUS and NASA-TLX. Success criteria will include feasibility, defined as achieving >70% recruitment and retention; usability, defined as an average SUS score >70; acceptable workload, defined as NASA-TLX scores within the low-to-moderate range; data quality, defined by completeness and internal consistency of collected measures; and scalability, indicated by minimal technical issues and positive user experience patterns across diverse settings.

Response accuracy, sensitivity, and specificity using both audio and video for the 99 children will produce continuous variables and will be analyzed using false positive and false negative counts to obtain sensitivity and specificity. Exploratory analysis will also be undertaken by subgroups in relation to motor type, severity, age, and gender where sufficient numbers are possible.

Exploratory supervised artificial intelligence and machine-learning approaches will be investigated using audio-only, video-only, and multimodal audio-video speech data collected through the My Voice Library platform. Potential approaches may include convolutional neural networks (CNN), recurrent neural networks (RNN), transformer-based architectures, automatic speech-recognition systems, lipreading models, and fine-tuning of existing large-scale speech-recognition models (eg, Whisper) [34]. Participant-specific speech-recognition systems may also be explored to support personalized communication approaches.

Labels for model development will be derived from the predefined target words and sentences embedded within the gamified modules based on the Frenchay Dysarthria Assessment–Second Edition, where the expected speech output is known a priori.

To minimize bias and data leakage, participant-level separation will be used between training, validation, and testing datasets. Cross-validation approaches will be applied where appropriate. Model performance will be evaluated using accuracy, sensitivity, specificity, false positive and false negative rates, and additional prediction performance metrics, as defined by the International Standards Organization [35].

As this is a protocol and feasibility-oriented research database study, analyses related to artificial intelligence and machine learning will be exploratory and intended to inform the future development of personalized speech-recognition systems rather than establish definitive clinical-performance benchmarks. Strategies to reduce overfitting will include the use of independent testing datasets, cross-validation procedures, and regularization or model-selection techniques where appropriate.

### Ethical Considerations

#### Ethics Approval

This research study will be conducted in full conformance with the principles of the “Declaration of Helsinki,” in particular through the maintenance of privacy and confidentiality of the participants, as well as informed consent through a Parent/Guardian Information Statement and Consent Form. This document states that participants understand their participation is completely voluntary and will not impact their relationship with the Cerebral Palsy Alliance, the Cerebral Palsy Alliance Research Institute, or the University of Sydney. It also states that participants can withdraw from the study at any time and that their personal information will be stored securely. The study has been approved by the University of Sydney’s Human Research Ethics Committee (2023/263).

#### Assessment and Documentation of Adverse Events

In this research study, the most likely adverse events could include participants feeling uncomfortable regarding their ability to finish the data collection or experiencing fatigue. This will be mitigated by providing the opportunity to pause and restart when they are ready.

Any participants who endorse statements reflecting an elevated risk of harm will be contacted by trained professionals on the investigator team to ensure they have appropriate support in place.

The researchers will communicate in writing any serious adverse events to The University of Sydney Human Research Ethics Committee [36]. As part of the annual reporting to The University of Sydney Human Research Ethics Committee, a summary report form [36] will also be uploaded to the University of Sydney’s ethics portal and will adhere to any recommendations made by the ethics committee in response.

#### Data Governance

Upon study completion, Cerebral Palsy Alliance will become the data custodian. When a researcher requests access to data (Data Requestor; Multimedia Appendix 2), these data access requests are reviewed by the My Voice Library research team to ensure that applications are complete, have the appropriate human research ethics approval, are feasible given the requested resources, have scientific validity, and are aligned with what the advisory panel has identified as priority research areas. If the proposed research project is considered detrimental to the people from whom the data originate, the data access may be denied or restricted. Upon approval from the My Voice Library research team, the data access request will be authorized for 12 months, with the ability to extend access for an additional 12 months.

#### Data Sharing

Deidentified personal data will be available to researchers, with current ethics approval from the University of Sydney’s Human Research Ethics Committee.

Upon completion of this research, all data will be deidentified where possible. However, audio and video data, by nature, cannot be 100% deidentified. Due to the nature of this database, data will be stored on REDCap and SharePoint in perpetuity, in accordance with the National Statement on Ethical Conduct in Human Research.

If a participant changes their mind about data sharing, the participant and/or their parents/carers can submit a request to the My Voice Library Research team for their data to be removed. While we have no control over the data that have already been shared through the My Voice Library, we will immediately enact the participant’s wishes and delete their data from the My Voice Library within 3 business days of receiving the request.

#### Dissemination Plans

The study results will be disseminated in published peer-reviewed journals and conference presentations. A summary of the findings will also be provided to participants by email IDs and published in the New South Wales CP Register [37] newsletters and the Bulletin of CP Quest [38], a world-leading collaboration, bringing together researchers and people with cerebral palsy, their families, carers, and advocates, as well as professional network channels.

All study investigators will be eligible for authorship. Authorship of publications and presentations will be decided based on the recommendations of the International Committee of Medical Journal Editors [39]. In order to qualify for authorship, investigators must meet each of the 4 criteria: Substantial contributions to the conception or design of the work; or the acquisition, analysis, or interpretation of data for the work; drafting the work or revising it critically for important intellectual content; final approval of the version to be published; and agreement to be accountable for all aspects of the work in ensuring that questions related to the accuracy or integrity of any part of the work are appropriately investigated and resolved.

General feedback on My Voice Library will be made available on the Cerebral Palsy Alliance Research Institute’s website and other channels that were used for recruitment. This website will also showcase any lay summaries from research studies that use the My Voice Library database. Researchers, clinicians, and engineers who access the My Voice Library data are required to provide information about publications and lay summaries of their research findings at the conclusion of their data access.

#### Data Management

All research participant data are stored, in perpetuity, on the AWS Cloud and the University of Sydney’s Data Management Storage. This includes any audio, video, or clinical data associated with the individual. All these data are deidentified. There are no hard copy materials of participant data stored for this project. My Voice Library data users (researchers, clinicians, and engineers who request to use the data) are stored on the University of Sydney’s REDCap server and SharePoint.

AWS data centers are housed in nondescript facilities, where physical access is strictly controlled. Access points at both the perimeter and at the building are monitored by staff using video surveillance, intrusion detection systems, and other electronic monitoring systems. Authorized staff must pass 2-factor authentication a minimum of 2 times to access the data center floors.

All data flowing across the AWS global network that interconnects their data centers and regions are automatically encrypted at the physical layer before data transit. The main risk to a database is a data breach. However, we take significant measures to ensure the safety of the data. In the event of a data breach or a suspected data breach, the My Voice Library Breach Response Plan will be executed.

#### Data Storage

All data flowing across the AWS global network that interconnects their data centers and regions are automatically encrypted at the physical layer before data transit.

All data will be managed in accordance with the University of Sydney Secure Research Data Store. Audio and video data will be stored in a number-coded deidentifiable format. Video data will, where possible, be deidentified using a privacy-enabling algorithm. The data will be accessible for the research team. The privacy-enabling algorithm prevents face recognition software from reliably recognizing faces while maintaining facial details in the recording [31]. Such deidentified personal data will be available to researchers, with current ethics approval from the University of Sydney’s Human Research Ethics Committee.

#### General Security

Access to My Voice Library will only be granted to bona fide researchers, clinicians, and engineers whose applications have been reviewed by the My Voice Library research team (where necessary). All data in My Voice Library are encrypted in-transit and at-rest. All web traffic is securely transferred over an HTTPS internet security protocol. All activities in the database are logged and tracked for compliance and security purposes. All data (including logs) are backed up daily. Annual external security audits and penetration testing are performed.

#### Data Retention and Archiving Process

My Voice Library is a research database—with the main aim of aggregating reidentifiable audio or video data from children with cerebral palsy and dysarthria aged 8 to 18 years. The data will be used in studies that have human research ethics approval, and individuals have consented to their nonidentified (but reidentifiable) data being shared. While these data will be used for the main purpose of the database, which enables data for real-time communication innovations, all requests to access the data in My Voice Library must include a summary of their project (to ensure it aligns with the objectives of this database) and evidence of their human research ethics approval or exemption.

#### Data Deletion

Only the My Voice Library research team has permission to delete data. If a participant wishes to remove data from the My Voice Library, they may submit a request (Multimedia Appendix 2). These requests will be actioned within 2 business days of receiving the request. However, these data will not be deleted from the daily backups. If a backup restore is required, all data erasure requests will be reviewed within 48 hours of the daily backup restore.

Participants can email the My Voice Library Research Team. This information is provided in the participation information sheet. Participant data will be deleted from active storage only. Previous daily backups will not be purged. If, for some reason, a systemwide data restore from a daily backup occurs after data have been deleted before the backup is overwritten, the My Voice Library data team will delete the participant data again. Any prior data access requests that included these data will not be contacted to delete the data.

#### Data Usage

The findings from this study will be published in peer-reviewed journals and presented at conferences. Only aggregated data will be reported.

Data collected for this study will be stored in perpetuity, as the purpose of the study is to allow future use of the data.

Therefore, for the third phase and beyond, when a researcher, clinician, or engineer requests access to nonidentifiable data (voice samples) stored on My Voice Library, they must include evidence of the following:

1. Human research ethics (or country-equivalent) approval for the original study in which the data will be used.
2. How they will disseminate the data.

#### Research Data

All data in My Voice Library are reidentifiable to the research team that manages the data and to them only. All results will either be shared and/or presented as a nonidentified individual case with limited clinical data or in an aggregated format.

#### Participant Clinical Data—My Voice Library

Participant clinical data include demographic data (birth year, sex, and country) and primary motor type of cerebral palsy; classifications of vision, speech, hearing, gross motor, fine motor, communication, eating, and drinking; and payment information. A comprehensive clinical record for each individual—using words and phrases—was obtained using the Frenchay Dysarthria Assessment 2.

#### Participant Compensation

The authors have a budget to pay for 10 people with lived experience of cerebral palsy and dysarthria, as well as clinicians and engineers who will join the advisory panel. Payment is Aus $50 (US $34.45) for each scheduled meeting during the study. The authors also have a budget to pay each participating child Aus $100 (US $68.90) for completed data collection activities.

#### Measures

These tools were selected based on the best available evidence; they are not timed, they do not require verbal responses, and they have features that are appropriate for accommodations via assistive technology access if needed. My Voice Library will be evaluated using usability indicators such as audio and video accuracy, sensitivity, and specificity. Prediction performance will be estimated to evaluate the artificial intelligence and machine-learning pattern, as defined by the International Standards Organization [32].

#### Recontact

The participating children and their families will be asked whether they would like to be contacted for future research projects aimed at developing real-time communication technology solutions. These contact details will be securely stored in REDCap and hosted outside the My Voice Library under the University of Sydney’s data management.

## Results

The National Health and Medical Research Council funded the project in April 2021. Data collection commenced in March 2024. As of April 2026, thirteen of 99 participants had been recruited at the time of submission of the paper. Preliminary data analysis commenced in 2026. The results of the preliminary analysis are expected to be published in 2027, and the full results are anticipated to be published once all 99 participants have been successfully recruited.

## Discussion

### Expected Outcomes

The establishment of My Voice Library is expected to enable significant advances in personalized communication technologies for children with cerebral palsy and dysarthria. By providing a high-quality, standardized repository of audio and visual speech samples, collected through gamified modules based on the Frenchay Dysarthria Assessment 2, the platform will support researchers, clinicians, and engineers to collaboratively develop and refine personalized speech-recognition models. These datasets and cross-disciplinary workflows are anticipated to improve the precision and responsiveness of real-time communication tools and ultimately enhance communicative participation for children with cerebral palsy by increasing the likelihood that their speech is understood in everyday contexts.

### Strengths and Limitations

Building large, representative databases of dysarthric speech presents several recruitment challenges that can limit both sample diversity and data quality. The population of interest is relatively small and heterogeneous, with wide variation in etiology, severity, co-occurring motor impairments, and communication access needs, making it difficult to recruit cohorts that reflect the full spectrum of dysarthric speech [1]. Many potential participants rely on caregivers, support workers, or clinical services to facilitate involvement, introducing logistical and consent-related barriers [3]. Families may also experience research fatigue or perceive limited direct benefit, reducing willingness to participate in time-intensive protocols.

Within this context, My Voice Library offers a structured approach to mitigating some of these challenges. It embeds high-quality voice and video assessments through gamified modules based on the *Frenchay Dysarthria Assessment 2* [28] and was co-designed with an active patient and a public involvement advisory group. This design enables the collection of speech data in controlled yet naturalistic environments, enhancing clinical relevance while supporting sustained engagement. The modular, gamified format reduces fatigue and increases both the quantity and diversity of data contributed by children. Additionally, the project’s information governance framework facilitates ethically approved research use of real-world data, providing families with clearer assurances around privacy, consent, and purpose.

### Conclusion

This project describes the development of My Voice Library, a repository of high-quality audio and visual speech datasets from children with cerebral palsy and dysarthria. By enabling researchers, clinicians, and engineers to contribute to and access shared datasets, the repository holds the potential to support the development of personalized speech-recognition technologies that facilitate real-time communication. If the anticipated outcomes are achieved, My Voice Library will provide a foundation for future research and innovation aimed at improving communicative participation for children and young people with cerebral palsy who have speech impairments.

## Supplementary material

10.2196/97614 Multimedia Appendix 1 Data dictionary.

10.2196/97614 Multimedia Appendix 2 My Voice Library research request form.

10.2196/97614 Checklist 1 GRIPP2 checklist.

10.2196/97614 Peer Review Report 1 Peer-review report from the National Health and Medical Research Council (NHRMC) Ideas Grant Committee, Australian Government.

## Abbreviations

AWS: Amazon Web Services
CNN: convolutional neural network
NASA TLX: NASA Task Load Index
REDCap: Research Electronic Data Capture
RNN: recurrent neural network
SUS: System Usability Scale
