# Supplementary material for: My Voice Library: Protocol for Developing Audio and Visual Datasets to Enable Personalized Real-Time Communication for People With Dysarthria
Source: JMIR Res Protoc. 2026 Jul 8;15:e97614. doi: 10.2196/97614 (PMC13345347; doi:10.2196/97614)
Supplement: Multimedia Appendix 1 [file resprot-v15-e97614-s001.docx]

| **Field Name** | **Category** | **Description** | **Value** | **Notes/Logic** |
| --- | --- | --- | --- | --- |
| Participant_ID | Mandatory | Unique ID for the participant | String, alphanumeric | One step removed from personal information. Can only be viewed by the Research Team and Data Custodians. |
| Participation_Information_Sheet |  | Participation Information Sheet | Downloadable document |  |
| Participant_Consent_Form |  | Participant Consent Form | Downloadable document |  |
|  |  | Name, first and last name |  |  |
| Consent | Mandatory | Do you provide consent? | 0=No  1=Yes |  |
| Demographic |  | Tell us a bit about yourself |  |  |
| Birth_year | Mandatory | What year were you born? | XXXX (years) | 1900+ [if person is younger than 8 years Stop no action] |
| Sex | Mandatory | What do you identify as [dropdown menu]? | 1=Male  2=Female  3=Intersex  4=Other |  |
| Country_residence | Mandatory | Do you currently live in Australia? | 0=No – go to ISO-3166 2-alpha code  1=Yes |  |
| Primary_motor | Mandatory | What is your predominant cerebral palsy motor type? | 1=Spastic 2=Dyskinetic - Dystonic 3=Dyskinetic - Choreoathetosis 4=Ataxic 5=Hypotonic |  |
| Visual_impairment | Mandatory | Do you have difficulty seeing things on a screen? [insert ‘Find 5 things’ picture list 10 items of which 5 are in the picture] | [if 5 correct items are picked] - 0= No  1=Yes – Stop actions | Participants who upon signing up to have their voice recorded on My Voice Library, do not pass the Screening Questionnaire as per items above, will be thanked and informed that they are at this time not eligible to be part of the study at this time. |
| VFCS | Mandatory | How would you describe your ability to see? [dropdown with descriptors] | 1=VFCS I 2=VFCS II 3=VFCS III 4=VFCS IV 5=VFCS V | Visual Function Classification System ([VFCS](https://onlinelibrary.wiley.com/doi/full/10.1111/dmcn.14270))© Score |
| Hearing_impairment | Mandatory | Do you have difficulty hearing? Click here and let us know if you can hear what is playing? [insert a short 30 seconds little happy music piece] | 0=No 1=Yes – Stop actions |  |
| GMFCS | Mandatory | How would you describe how you are moving? [dropdown pictogram of the different levels] | 1=GMFCS I 2=GMFCS II 3=GMFCS III 4=GMFCS IV 5=GMFCS V | Gross Motor Functioning Classification System ([GMFCS](https://canchild.ca/en/resources/42-gross-motor-function-classification-system-expanded-revised-gmfcs-e-r))© Score |
| MACS | Mandatory | How would you describe how you are able to use your fingers and hands to hold and move things? [dropdown with descriptors] | 1=MACS I 2=MACS II 3=MACS III 4=MACS IV 5=MACS V | Manual Ability Classification System ([MACS](https://www.macs.nu/))© Score |
| CFCS | Mandatory | How would you describe how other people are able to understand what you are saying? [dropdown with descriptors] | 1=CFCS I 2=CFCS II 3=CFCS III 4=CFCS IV 5=CFCS V | Communication Function Classification System ([CFCS](http://cfcs.us/wp-content/uploads/2018/11/CFCS_English_CP.pdf))© Score |
| VIKING | Mandatory | How would you describe how your speech is? [dropdown with descriptors] | 1=Score I 2=Score II 3=Score III 4=Score IV | [Viking Speech Scale, 2010©](https://eu-rd-platform.jrc.ec.europa.eu/sites/default/files/Viking-Speech-Scale-2011-Copyright_EN.pdf) |
| EDACS | Mandatory | How would you describe your ability to eat and drink? [dropdown with descriptors] | 1=EDACS I 2=EDACS II 3=EDACS III 4=EDACS IV 5=EDACS V | Eating and Drinking Ability Classification System for Individuals with Cerebral Palsy (EDACS) © Score |
| Payment data | Not mandatory |  |  | Link to REDCap database |
|  |  | Payment Participants who are part of the My Voice Library Study, will be offered: An eGift of Digital Mastercard $100 for contributing time. |  |  |
|  |  | I agree to be paid $100 for participating in the My Voice Library Study. | 0=No  1=Yes  Required 1  Stop actions on 0 |  |
|  | Mandatory | Role: Research Participant |  | Show the field ONLY if: [agree] = '1' |
|  | Mandatory | First name: | text, Required |  |
|  | Mandatory | Last name: | text, Required |  |
|  | Mandatory | Date: | text (date_dmy), Required |  |
|  |  | To enable processing of this payment please fill out the below details: |  |  |
|  | Mandatory | Email: | text, Required |  |
|  |  | Confirm Email: | text, Required |  |
| My Voice Library |  |  |  | Participants who upon setting up their account on My Voice Library and where the system recognises that they audio and/or video quality is too limited for a successful recording. The participant will be given information on possible options on how to improve the audio and video quality. |
|  | Mandatory | Before we start, please confirm your account | text, Required |  |
|  | Mandatory | Almost there – Submit an audition |  |  |
|  | Mandatory | Welcome to My Voice Library!  Before we start your recording session, please review this checklist to make sure you are ready:   - I’m in a completely quiet room - I’m using a headset microphone to record - I’m using…….browser - I’m using a laptop or desk top computer   Once you have ticked all requirement, click NEXT | [Requirements for browser?] |  |
|  | Mandatory | Let’s make sure your microphone is accessible. On the screen, you may be asked to allow My Voice Library access to your microphone. Be sure to click ‘Allow’ so we can receive your audio!  When you are ready, click NEXT. |  |  |
|  | Mandatory | Background noise check  Next, click RECORD to start recording your background noise level. My Voice Library will automatically record sound for 3 seconds. Remember to keep quiet as it counts down! |  |  |
| Introduction to activities in My Voice Library – set up |  | In order to deliver your recording content that is interesting and unique, we would like to ask you a few questions [theme and questions to be generated in upcoming workshop]. | - Face tracker algorithm – trim image around the lips for future lip reading but de-identify the rest of the face. Video stored on secure server – check landmark detection before processing. |  |
| Non-words [example] | Mandatory | Can you say: /iy-p-ah/ | Face tracker algorithm, face landmark extraction | 5 repetitions of non-words |
| Lips: at rest [example] | Mandatory | [Just relax, don't smile, try and breath through your nose and look into the camera for 3 seconds] | Face tracker algorithm, face landmark extraction | - Repeat 5 times |
| Palate: Maintenance  [example] | Mandatory | Can you say ‘ah-ah-ah’ | Face tracker algorithm, face landmark extraction | - Repeat 5 times |
| Laryngeal: Time [example] | Mandatory | Can you say ‘ah’ for as long as possible | Face tracker algorithm, face landmark extraction |  |
| Intelligibility: Words [example] | Mandatory | Say: [pet; people; map] | Face tracker algorithm, face landmark extraction | - Repeat 2 times |
| Intelligibility: Sentences [example’ | Mandatory | Say: [I’ve got a new toy] | Face tracker algorithm, face landmark extraction | - Repeat 2 times |
| Celebrations of finishing the activities |  | Celebrations! |  |  |
| Invitation text to future studies |  |  |  |  |
| To be contacted for future research | Not mandatory | I agree to be contacted for future research. |  | Link to REDCap database |
|  | Mandatory | First name: | text, Required |  |
|  | Mandatory | Last name: | text, Required |  |
|  | Mandatory | Email: | text, Required |  |
|  | Mandatory | Phone Number: | text, Required |  |
|  | Mandatory | Date: | text (date_dmy), Required |  |
|  | Mandatory | Signature: | file (signature), Required |  |
