# Supplementary material for: My Voice Library: Protocol for Developing Audio and Visual Datasets to Enable Personalized Real-Time Communication for People With Dysarthria
Source: JMIR Res Protoc. 2026 Jul 8;15:e97614. doi: 10.2196/97614 (PMC13345347; doi:10.2196/97614)

Request for Assistance with Voice samples for Research from My Voice Library database

Date:

Project title:

Person/s submitting:

Position/s:

Name(s) of supervisors if a student:

Place of work and other affiliations:

Contact details:

Describe this project and the potential benefits of this project for people with cerebral palsy?

What evidence is available to support the need for this research?
For example: discussions with peers/service users, results of other research, literature etc)

Describe the involvement of people with lived experience in the design and development of this project.

What impact will this project have on the academic world?

What data are you requesting? (if applicable)

What assistance with recruitment are you requesting? (if applicable)
For example: one dedicated email to potential participants (children with CP age 6-12 years, GMFCS levels III-V, who live in metropolitan Sydney) with a link to the survey plus advertisement in one CP Register newsletter)

Please provide your ethics approval from your institution:

☐ I agree to acknowledge the contribution of the My Voice Library to our study in publications and presentations.

☐ I agree to provide a timely lay summary of the study findings so that results can be shared with current and future participants of the My Voice Library in forthcoming My Voice Library newsletters.

☐ I understand that the information I have provided on this form will be considered by My Voice Library staff, and may be considered by other relevant experts. In this process every care will be taken to protect both my privacy and the intellectual property in relation to this project.

Name: **Signed:**

Ethical oversight

The ethical aspects of My Voice Library are overseen by the University of Sydney **[INSERT approval number once approval is obtained].** As part of this process, My Voice Library is conducted in accordance with the National Statement on Ethical Conduct in Human Research (2007).

The Manager, Ethics Administration, The University of Sydney
Telephone: +61 2 8627 8176
Email: human.ethics@sydney.edu.au
Fax: +61 2 8627 8177 (Facsimile)

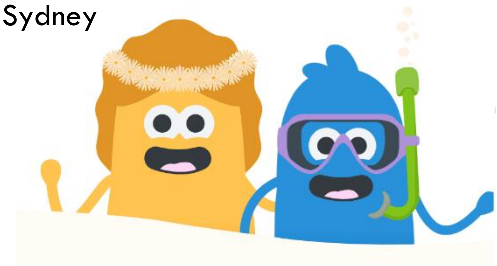

Supplement: Multimedia Appendix 2 [file resprot-v15-e97614-s002.pdf]
