# Supplementary material for: My Voice Library: Protocol for Developing Audio and Visual Datasets to Enable Personalized Real-Time Communication for People With Dysarthria
Source: JMIR Res Protoc. 2026 Jul 8;15:e97614. doi: 10.2196/97614 (PMC13345347; doi:10.2196/97614)
Supplement: Checklist 1 [file resprot-v15-e97614-s003.pdf]

GRIPP2 Reporting Checklist – Short Form

| **Section and topic** | **Item** | **Section where reported and details** |
|---|---|---|
| 1. Aim | Report the aim of patient and public involvement (PPI) in the study. | Engagement of people with lived experience of cerebral palsy and dysarthria in this research was essential to ensure that patient perspectives were integral to preparation, execution and translation. This is outlined in the section Patient and public involvement. |
| 2. Methods | Provide a clear description of the methods used for PPI in the study. | People with lived experience of cerebral palsy and dysarthria have been involved throughout the development of My Voice Library. This study will use an Advisory Panel, comprising people with cerebral palsy and dysarthria, carers, and researchers which has and will provided guidance, ideas, and networks. Advisors have shaped the format, language, and content of the modules, information materials, and questionnaires to ensure they reflect stakeholder priorities. This is outlined in the section Patient and public involvement. |
| 3. Study results | Outcomes—Report the results of PPI in the study, including both positive and negative outcomes. | A team member with lived experience also appears in an information video and will be involved in disseminating study findings. This is outlined in the section Patient and public involvement. |
| 4. Discussion and conclusion | Outcomes—Comment on the extent to which PPI influenced the study overall. Describe positive and negative effects. | One investigator with lived experience, along with additional parents and young people, contributed feedback on the platform design, module burden, and participant materials. This is outlined in the section Patient and public involvement. |

My Voice Library: Protocol and research database of audio and visual datasets to enable personalised real-time communication for people with dysarthria

| | | |
|---|---|---|
| 5. Reflections/clinical perspective | Comment critically on the study, reflecting on the things that went well and those that did not, so others can learn. | The concept for My Voice Library emerged directly from conversations with people seeking faster, more reliable communication tools. This is outlined in the section Patient and public involvement. |
