# Supplementary material for: My Voice Library: Protocol for Developing Audio and Visual Datasets to Enable Personalized Real-Time Communication for People With Dysarthria
Source: JMIR Res Protoc. 2026 Jul 8;15:e97614. doi: 10.2196/97614 (PMC13345347; doi:10.2196/97614)
Supplement: Peer Review Report 1 [file resprot-v15-e97614-s004.pdf]

Australian Government
National Health and Medical Research Council

NHMRC

Application Assessment Summary

2021 Ideas Grants

Application ID: 2012882

Chief Investigator A: Doctor Petra Karlsson

Administering Institution: University of Sydney

Table 1: Summary of the assessment of your application against the 2021 Ideas Grants Assessment Criteria.

Criteria			
	1	Research Quality (35%)	5.000
	2	Innovation and Creativity (25%)	5.000
	3	Significance (20%)	5.500
	4	Capability (20%)	6.250
		**Overall Score**	**5.350**
		**Category**	**5**

The funding cut-off for 2021 Ideas Grants applications fell at approximately 5.600.

Table 2: **Overview of all applications.**

Category and average scores for each of assessment criteria is provided below.

The **overall funded rate** for **2021 Ideas Grants** applications was 9.5%.

Category	No. of applications	**Research Quality (35%)**	**Innovation and Creativity (25%)**	**Significance (20%)**	**Capability (20%)**
6	366	5.578	5.675	5.651	5.979
5	1741	4.825	4.991	5.026	5.422
4	472	3.940	4.177	4.278	4.680
3	30	2.904	3.171	3.322	3.623
2	1	2.400	2.200	2.200	2.400
1	1	1.000	1.600	1.400	1.800

Table 3: **Peer Reviewer comments**
Qualitative feedback on your application is provided below in the form of Peer Reviewer comments.

Comments from peer reviewers are provided verbatim, in accordance with the intent of NHMRC's independent expert peer review processes. Comments reflect the assessment of individual peer reviewers and are provided to support applicants' understanding of how their application was scored.

NHMRC will respond to complaints about the peer review process but not about the merits of peer reviewer comments. Applicants are encouraged to speak to their Institutional RAO if they are concerned about peer review outcomes. For more information on the steps NHMRC has taken to support quality peer review in the Ideas Grant scheme, visit:

https://www.nhmrc.gov.au/funding/peer-review/disclaimer

**Peer Reviewer Comments:**
**Stage:** Peer Reviewer Assessment<br>**Assessor Role:** Peer Reviewer<br>**Question:** Research Quality (35%)<br><br>The proposal is comprehensive. I wondered if 9 school children would be enough. How will assessments be made as to whether the child has mild/moderate/severe dysarthria and also why 6 sessions were chosen in phase 1 of the study.
**Stage:** Peer Reviewer Assessment<br>**Assessor Role:** Peer Reviewer<br>**Question:** Research Quality (35%)<br><br>Develop a repository of high-quality of audio and visual datasets- develop the building blocks to establish technological solutions for children with dysarthria . The three phases of the research plan are well mapped out.
**Stage:** Peer Reviewer Assessment<br>**Assessor Role:** Peer Reviewer<br>**Question:** Research Quality (35%)<br><br>The project is well-described and includes a sound rationale as well as clearly defined aims. The methodological approach is mapped out nicely. I found it hard to ascertain whether the MY Voice Library had already been developed and there was preliminary data to support the potential success of this project, or whether the project was only at the conception phase. Great that you have included advisory panel of people with lived experience of CP in the project, but I imagine that there would be significant benefit to including them in the design phase at the start of the project rather than just the testing phase.
**Stage:** Peer Reviewer Assessment<br>**Assessor Role:** Peer Reviewer<br>**Question:** Research Quality (35%)<br><br>The project aims and proposed research plan are supported by a very well justified rationale well-defined, very highly coherent and have a very good study design and approach. The plan and aims would be likely to be competitive with high quality, similar research proposals internationally. Scientific and technical risks are well addressed

Stage: Peer Reviewer Assessment
Assessor Role: Peer Reviewer
Question: Innovation and Creativity (25%)

The investigators have made it clear that although there is work in progress, the proposal they have put forward is a significant advancement from what is currently available.

Stage: Peer Reviewer Assessment
Assessor Role: Peer Reviewer
Question: Innovation and Creativity (25%)

The team has identified a gap which needs to be addressed in order to progress innovative communication biotechnologies for children with CP.

Stage: Peer Reviewer Assessment
Assessor Role: Peer Reviewer
Question: Innovation and Creativity (25%)

The concept is highly innovative. I would have liked some more information about how the library would be used by people with CP. what would the interface look like? would you need to develop this also?

Stage: Peer Reviewer Assessment
Assessor Role: Peer Reviewer
Question: Innovation and Creativity (25%)

The planned research demonstrates very highly innovative project aims, which will result in a very substantial impact on the research area.

Stage: Peer Reviewer Assessment
Assessor Role: Peer Reviewer
Question: Significance (20%)

This is an important area of research and the description of the problem is well described. The need to bridge communication difficulties and restore relationship building capacity in these young children is paramount.

Stage: Peer Reviewer Assessment
Assessor Role: Peer Reviewer
Question: Significance (20%)

Well-outlined and argued.
• the most common disability of childhood with 50% having speech impairments
• limitations of existing communication technologies even with mild dysarthria
• no voice repository accessible for research and critical technology innovation in Australia, none available for children world-wide
• technological advances are moving rapidly with AI and innovative technologies but high-quality datasets are needed for this development and evaluation.
• CLC treatment continuum where there is no chronic disease program specifically targeting CLC in this population is appreciated.
Outcomes, future research and sustainability are addressed.

Stage: Peer Reviewer Assessment
Assessor Role: Peer Reviewer
Question: Significance (20%)

This project has high significance as it addresses an important challenge in the field of disability.

Stage: Peer Reviewer Assessment
Assessor Role: Peer Reviewer
Question: Significance (20%)

The planned research will address an issue of considerable importance to advance the research. The outcome of the research may lead to significant outcomes for end users (people with CP), however, this has not been well described.

Stage: Peer Reviewer Assessment
Assessor Role: Peer Reviewer
Question: Capability (20%)

The team has all the expertise and skills required to successfully carry out this program of work. They have led a lot of the pilot work in this area.

Stage: Peer Reviewer Assessment
Assessor Role: Peer Reviewer
Question: Capability (20%)

The multi-disciplinary team brings relevant experience and expertise to the application and includes an appropriate consumer. CIA has the clinical and research expertise, a good track record and established international collaborations in this area. CIB brings bioengineering technical and medical device commercialisation expertise to the application.

Stage: Peer Reviewer Assessment
Assessor Role: Peer Reviewer
Question: Capability (20%)

The CIA demonstrates good capability to lead the team in achieving the project aims. The team appears well balanced and has access to the required resources and participants.

Stage: Peer Reviewer Assessment
Assessor Role: Peer Reviewer
Question: Capability (20%)

The CIA demonstrates a very strong capability to lead the team in achieving the project aims. Overall, the CIA team has exceptional capability to execute the project and deliver outcomes and has access to outstanding technical resources and infrastructure
